# *Aspergillus* Thyroiditis: A Complication of Respiratory Tract Infection in an Immunocompromised Patient

**DOI:** 10.1155/2013/741041

**Published:** 2013-12-18

**Authors:** Madiha M. Alvi, David S. Meyer, Nicholas J. Hardin, James G. deKay, Annis M. Marney, Matthew P. Gilbert

**Affiliations:** ^1^Department of Medicine, Division of Endocrinology and Diabetes, The University of Vermont College of Medicine, Burlington, VT 05401, USA; ^2^Department of Medicine, The University of Vermont College of Medicine, Burlington, VT 05401, USA; ^3^Department of Pathology and Laboratory Medicine, The University of Vermont College of Medicine, Burlington, VT 05401, USA

## Abstract

A 59-year-old male with past medical history significant for non-Hodgkin's lymphoma status after chemotherapy presented with acute onset of neck pain, odynophagia, and dysphagia associated with subjective fever, chills, and dyspnea. Physical findings included a temperature of 38.4°C, hypertension, and tachycardia. Patient was found to have anterior neck tenderness. Laboratory evaluation revealed neutropenia. The patient was started on empiric antibacterial and antiviral therapy and continued on home prophylactic antifungal treatment. Thyroid function tests revealed overt hyperthyroidism. A thyroid ultrasound showed heterogeneous echotexture without discrete nodules. Subacute thyroiditis was treated with methylprednisolone, metoprolol, and opiate analgesics. Patient's antibacterial, antifungal, and antiviral treatments were broadened. A fine needle aspiration was not conducted. The patient's condition deteriorated rapidly over his brief hospital course and he expired. Autopsy showed fungal thyroiditis secondary to disseminated invasive *Aspergillus*. This report describes the presentation of fungal thyroiditis secondary to disseminated invasive *Aspergillus* originating from the respiratory tract. The authors review the diagnostic challenges, pathophysiology, and treatment of this condition.

## 1. Introduction

*Aspergillus* thyroiditis (AT) remains the most commonly reported fungal thyroiditis, followed by *Candida* species [1]. It has primarily been a postmortem diagnosis in immune-compromised patients [2–4]. The diagnostic strategy in such patients remains a challenge. Fine needle aspiration (FNA) with subsequent cytology and culture remains the recommended, most frequent and most successful method used to diagnose AT [5, 6]. However, the utility of subjecting patients to invasive procedure such as FNA in the setting of critical illness remains unclear.

## 2. Case Presentation 

The patient is a 59-year-old male with past medical history significant for non-Hodgkin's lymphoma (NHL) (Stage IV Splenic Marginal Zone) and transfusion-dependent myelodysplasia. The patient was diagnosed with NHL seven years prior to presentation. NHL management included failed resection of spleen, inadequate response to rituximab, and eventual complete response to six cycles of fludarabine. The patient developed persistent pancytopenia and multilineage dysplasia. One year prior to presentation, the patient was admitted for disseminated herpes zoster infection (CN V1, ophthalmic involvement, and wrist/knee vesicles). One month prior to presentation, the patient tolerated a short course of decitabine.

At presentation, the patient complained of a three-day history of neck pain, odynophagia, and dysphagia with associated subjective fever and chills, dyspnea, fatigue, and loss of appetite. Physical findings included a temperature of 38.4°C, BP of 186/84, tachycardia with rate of 138 beats per minute, respiratory rate of 20 per minute, O_2_ saturation of 96% on room air, tongue with superficial white layer, and exquisite anterior neck tenderness. Laboratory evaluation revealed neutropenia with an absolute neutrophil count of 0.10 K/cmm (2.20–8.85 K/cmm), hemoglobin of 10.0 gm/dL (13.8–17.3 gm/dL), hematocrit of 28.2% (39.5–50.2%), platelets of 20 K/cmm (141–320 K/cmm), albumin of 2.6 g/dL (3.4–4.9 g/dL), TSH of 0.02 *μ*lU/mL (0.35–5.00 *μ*IU/mL), free T4 of 3.5 ng/dL (0.8–1.8 ng/dL), total T4 of 13.1 *μ*g/dL (4.5–10.9 *μ*g/dL), total T3 of 117 ng/dL (60–181 ng/dL), and free T3 of 2.5 pg/mL (2.3–4.2 pg/mL). A chest X-ray showed diffuse patchy opacities interpreted as pneumonia.

The patient was started on cefepime (2 grams IV Q 8 hrs) and vancomycin (1 gram Q 12 hrs), with continuation of home prophylactic fluconazole (100 mg PO QD). To further evaluate the patient's chief complaint of neck pain in the setting of abnormal thyroid studies, persistent tachycardia, and dyspnea, a thyroid ultrasound was done which showed heterogeneous low echogenicity without discrete nodules bilaterally, consistent with acute thyroiditis versus infiltrative thyroid neoplasm. Follow-up computerized tomography scan of neck, abdomen, and pelvis showed symmetric enlargement of the thyroid (normal shape, homogenous low attenuation) consistent with suspected subacute thyroiditis. Patchy lung opacities, right upper and middle lobe airspace consolidation with bilateral scattered nodules, and mediastinal and paraesophageal lymphadenopathy most consistent with fungal infection were also noted. Subacute thyroiditis was treated with methylprednisolone (40 mg IV Q 6 hrs), metoprolol (5 mg IV Q 6 hrs), and opiate analgesics. Patient's antibiotics were broadened to include voriconazole (6 mg/kg IV Q 12 hrs), meropenem (1 gram Q 8 hrs), vancomycin (1500 mg Q 12 hrs), and valacyclovir (500 mg daily).

The patient's condition deteriorated rapidly over hospital days 3 and 4, with progression of dyspnea, onset of cheyne-stokes breathing, acute delirium, and severe anterior neck pain necessitating opiate PCA (patient controlled analgesia). Although the endocrinology consultants strongly considered a FNA for diagnosis, the management plan (i.e., empiric antibacterial and antifungal therapies) would not have changed based on cytopathology. Furthermore, the procedure put the patient at risk of both discomfort and hemorrhage. On hospital day four, patient, family, and treatment team moved to comfort-directed care. The patient expired on day 4 of hospitalization.

At autopsy, there was no residual lymphoma and no residual myelodysplastic syndrome. There was bone marrow damage thought to be due to treatment effect. The lungs showed bilateral pulmonary hemorrhagic lesions which proved to be aspergillosis. There were numerous fungal hyphae in multiple sites, including the lungs, liver, right adrenal gland, and left kidney. In the right common iliac vein there was a thrombus which was infected with the hyphae, and some of this material had embolized to the lungs (right hilar embolus).

**Figure 1:**
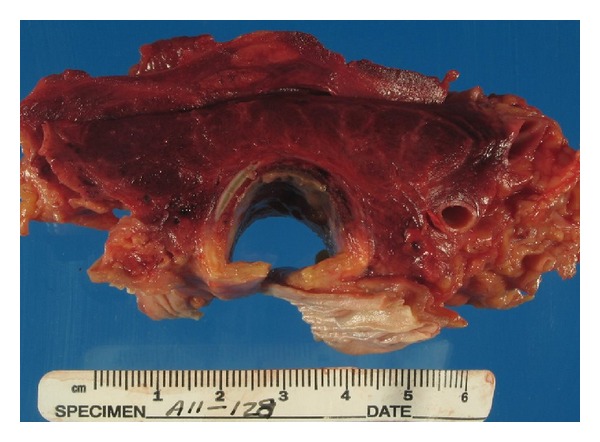
Autopsy gross digital image of cross section of trachea (bottom) surrounded by hemorrhagic thyroid gland and soft tissue. The thyroid and strap muscles are intensely hemorrhagic due to infarction from the vascular involvement by fibrin thrombi infected with the *Aspergillus* hyphae.

**Figure 2:**
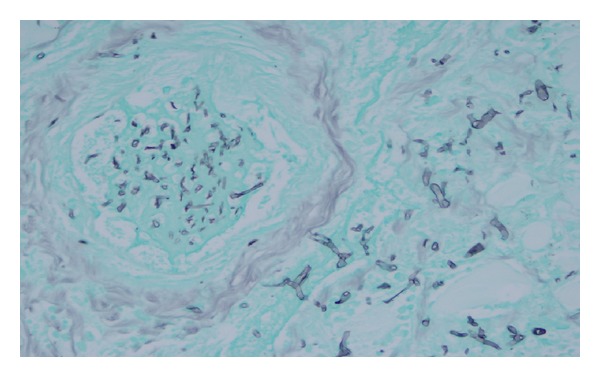
Image of thyroid at autopsy showing thrombus with hyphae in vessel (left) and invasive *Aspergillus* organisms in thyroid (right). Gomori Methenamine Silver stain, 60x objective.

**Figure 3:**
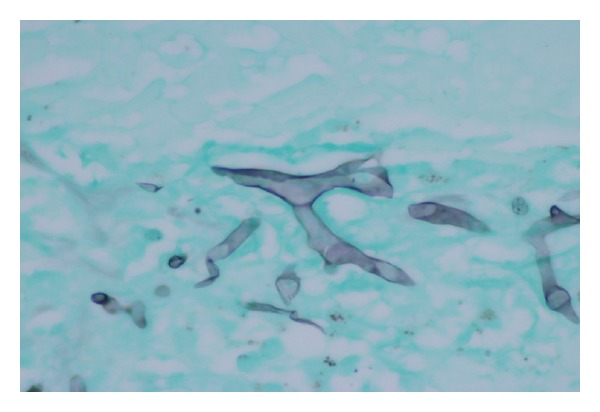
This image shows several hyphae, exhibiting the typical 45 degree branching and septate forms characteristic of *Aspergillus*. Gomori Methenamine Silver stain, 60x objective.

The trachea had a large ulcerating lesion covered with yellow-green exudate centered on the anterior wall, which on examination had penetrated into and involved the thyroid. The thyroid was intensely and diffusely hemorrhagic in the gross specimen (see Figure 1). Microscopically, there was hemorrhagic infarction of the thyroid and surrounding soft tissue, with ischemic necrosis, numerous fibrin thrombi containing fungal elements, hemorrhage, and numerous fungal hyphae typical of those seen in *Aspergillus*, that is, branching septate hyphae, about 2 to 7 microns in diameter, branching at 45 degrees (see Figures 2 and 3). The fungal morphology was confirmed by Gomori Methenamine Silver stain.

## 3. Discussion

*Aspergillus* thyroiditis is the most commonly reported fungal thyroiditis, followed by *Candida* species [1]. Other fungal organisms reported are *Histoplasma capsulatum*, *Cryptococcus neoformans*, and *Coccidioides immitis* [1]. The first report in the medical literature of *Aspergillus* infection involving the thyroid gland was by Grekin et al. in 1950. *Aspergillus* thyroiditis (AT) has primarily been a postmortem diagnosis in immunocompromised patients [2–4]. Hori et al.'s review of 107 autopsy records of adult patients with disseminated invasive aspergillosis (DIA) found thyroid involvement in 13 patients (12%) [7].

*Aspergillus* thyroiditis is most often described in the setting of widespread disseminated infection in immunocompromised patients, such as in those with acquired immunodeficiency syndrome, leukemia, solid organ transplantation, bone marrow transplantation, autoimmune diseases, or pharmacological immunosuppression [1]. Boon et al. reported eight cases of thyroid abscesses among 32 patients with fatal aspergillosis [8]. Amongst immunocompromised patients, multiple cases of AT have been reported in renal transplant patients [9–13].

*Aspergillus* species has angioinvasive properties and disseminates via hematogenous spread. Mechanisms include neutrophil recruitment, activation of cellular immunity, inhibition of host defense (NADPH oxidase and macrophages), and suppressed T-cell response, all leading to secondary inflammation [14]. The thyroid gland is usually described as relatively resistant to infection owing to its rich lymphatic drainage, high iodine content, and distinct fibrous capsule. Nonetheless, it is a frequent site of dissemination in invasive aspergillosis due to its rich blood supply and due to aggressive vascular invasion associated with *Aspergillus* species progressing to infection across tissue planes. The lung is a common portal of entry for *Aspergillus* spores with subsequent spread to the central nervous system, liver, spleen, gut, adrenal glands, and skin. In our case, it appears that the thyroid involvement was due to direct invasion from a tracheal ulcer infected with *Aspergillus*. Extrapulmonary involvement occurs with advanced stages of invasive aspergillosis and represents an ominous sign.

*Aspergillus* thyroiditis often presents with acute neck pain along with signs and symptoms of thyrotoxicosis [15]. Local signs and symptoms of infection are indistinguishable from other types of infectious thyroiditis and include fever, anterior cervical pain, and thyroid enlargement sometimes associated with dysphagia and dysphonia [1]. This makes it difficult to distinguish from subacute or suppurative thyroiditis. Patients with more chronic thyroid infections often have bilateral disease. Thyroid lesions in cases of DIA have been described as focal abscesses, patchy hemorrhagic lesions due to vascular invasion, or diffuse necrotizing thyroiditis [16, 17]. Diagnosis is delayed in most cases of AT and is frequently revealed at autopsy. A comprehensive review by Denning and Stevens reported that 9–15% of patients with DIA had thyroid involvement at autopsy [18].

Survival of patients with DIA depends on early recognition and prompt initiation of therapeutic measures. However, invasive procedures are often not feasible in patients with hematological malignancies, thus further delaying prompt identification of DIA. It is suggested that when fungal thyroiditis is suspected, one could proceed with ultrasonography, followed by needle aspiration and prompt antifungal treatment, if it has not been already started. Ultrasound remains the best way to evaluate for possible abscess formation in patients with AT [19]. Fine needle aspiration (FNA) with subsequent cytology and culture remains the most frequent and most successful method used to diagnose AT [5, 6]. Thyroid uptake and scan have also been shown to aid in the diagnosis of AT [20]. Further information in case of disseminated disease is usually available through blood or other body fluid cultures. Serum galactomannan antigen testing has been advocated for evaluating patients with disseminated *aspergillus* infection [21, 22].

Treatment of isolated fungal infection in thyroid gland and/or DIA is based on IDSA (Infectious Disease Society of America) guidelines for treating fungal infections [23]. These guidelines do not specify any preferred or different line of treatment for *aspergillus* thyroiditis. Triazoles (especially voriconazole) remain the drug of choice [24]. Denning et al. published data in 1990 and 1996, which showed that all untreated patients with invasive aspergillosis expired, and, likewise, all those treated for <8 days expired. The time of initiation of appropriate antifungal therapy is critical for all patients, but it is particularly so for those whose immunodeficiency (e.g., neutropenia) will not be resolved. As failure rates are high, switching the antifungal therapy early when the first treatment choice fails may be important [18, 25].

In our case, the patient was already being treated with broad spectrum antibiotics including antifungal therapy given his immunocompromised status and lung finding on computerized tomography most consistent with fungal infection. FNA of thyroid at this point would not have changed the clinical management, was invasive, and presented additional bleeding risk, and, thus was not pursued. The patient's clinical presentation was consistent with thyroiditis and as confirmed on autopsy report the presence of disseminated *aspergillus* infection, the etiology was *aspergillus* thyroiditis. Treatment for both DIA and AT remains the same. Clinical debate over the utility of invasive diagnostic procedures, such as FNA, often focuses on obtaining an accurate diagnosis. However, failure to impact clinical management remains a solid criterion for not exposing patients to invasive diagnostic procedures.
